# The Added Value of Transcatheter CT Hepatic Angiography (CTHA) Image Guidance in Percutaneous Thermal Liver Ablation: An Experts’ Opinion Pictorial Essay

**DOI:** 10.3390/cancers16061193

**Published:** 2024-03-18

**Authors:** Robbert S. Puijk, Madelon Dijkstra, Susan van der Lei, Hannah H. Schulz, Danielle J. W. Vos, Florentine E. F. Timmer, Bart Geboers, Hester J. Scheffer, Jan J. J. de Vries, Maarten L. J. Smits, Rutger C. G. Bruijnen, Frédéric Deschamps, Thierry de Baère, Bruno C. Odisio, Martijn R. Meijerink

**Affiliations:** 1 Department of Radiology & Nuclear Medicine, Amsterdam UMC, Vrije Universiteit Amsterdam, 1081 HV Amsterdam, The Netherlandsmr.meijerink@amsterdamumc.nl (M.R.M.); 2 Cancer Center Amsterdam, 1081 HV Amsterdam, The Netherlands; 3 Department of Radiology & Nuclear Medicine, OLVG, 1091 AC Amsterdam, The Netherlands; 4 Department of Radiology & Nuclear Medicine, Garvan Institute of Medical Research, Kinghorn Cancer Centre, Darlinghurst, NSW 2010, Australia; 5 Department of Radiology & Nuclear Medicine, Noordwest Ziekenhuisgroep, 1815 JD Alkmaar, The Netherlands; 6 Department of Radiology & Nuclear Medicine, UMC Utrecht, 3584 CX Utrecht, The Netherlands; 7 Departement d’Anesthésie, de Chirurgie, et de Radiologie Interventionnelle, Gustave Roussy, Université Paris-Saclay, 94805 Villejuif, France; 8 Department of Interventional Radiology, Unit 1471, The University of Texas MD Anderson Cancer Center, Houston, TX 77030-4009, USA

**Keywords:** liver tumor, thermal liver ablation, image guidance, computed tomography (CT), CT hepatic arteriography (CTHA), microwave ablation (MWA), radiofrequency ablation (RFA), quality control

## Abstract

**Simple Summary:**

Image-guided tumor ablation has become an indispensable part of oncological care, with an increasing demand and need for novel implements to optimize treatment outcomes. The purpose of this pictorial essay was to illustrate the added value of intra-arterial contrast administration during CT-guided liver tumor ablation procedures. Eight clinical cases from three tertiary referral institutions demonstrate the ability to improve the conspicuity of the target liver tumor(s) and identify surrounding critical vascular structures, promptly detect ‘vanished’ and/or additional tumors, differentiate local tumor progression from non-enhancing scar tissue, and instantly detect and respond to iatrogenic hemorrhagic events. In conclusion, although at the cost of adding a minor but safe intervention, the use of an intra-arterial-administered contrast agent during a CT-guided thermal ablation procedure is a potential quality-improving real-time image-guiding method and should therefore be embedded in (inter)national standards of practice.

**Abstract:**

With the rapidly evolving field of image-guided tumor ablation, there is an increasing demand and need for tools to optimize treatment success. Known factors affecting the success of (non-)thermal liver ablation procedures are the ability to optimize tumor and surrounding critical structure visualization, ablation applicator targeting, and ablation zone confirmation. A recent study showed superior local tumor progression-free survival and local control outcomes when using transcatheter computed tomography hepatic angiography (CTHA) guidance in percutaneous liver ablation procedures. This pictorial review provides eight clinical cases from three institutions, MD Anderson (Houston, TX, USA), Gustave Roussy (Paris, France), and Amsterdam UMC (Amsterdam, The Netherlands), with the intent to demonstrate the added value of real-time CTHA guided tumor ablation for primary liver tumors and liver-only metastatic disease. The clinical illustrations highlight the ability to improve the detectability of the initial target liver tumor(s) and identify surrounding critical vascular structures, detect ‘vanished’ and/or additional tumors intraprocedurally, differentiate local tumor progression from non-enhancing scar tissue, and promptly detect and respond to iatrogenic hemorrhagic events. Although at the cost of adding a minor but safe intervention, CTHA-guided liver tumor ablation minimizes complications of the actual ablation procedure, reduces the number of repeat ablations, and improves the oncological outcome of patients with liver malignancies. Therefore, we recommend adopting CTHA as a potential quality-improving guiding method within the (inter)national standards of practice.

## 1. Introduction

As thermal ablation, and especially the percutaneous approach, has become more popular and available in the treatment of primary and secondary liver malignancies over the last few years, optimizing treatment efficacy is one of the main goals ahead of us [1,2,3,4,5,6]. An adequate safety (or ‘peri-ablational’) margin, reflecting the distance from the initial lesion boundaries to the border of the post-treatment ablation zone, is one of the most determining factors influencing local tumor control following thermal ablation [7,8]. Circumferential safety margins of at least 5 mm, and preferably > 10 mm, are known to improve local control, respectively, with around 15% and 5% local tumor progression (LTP) rates during follow-up [7,9,10]. Complete ablation, commonly expressed as technical success rates or local tumor progression-free survival (LTPFS), should be pursued in all patients, and confirmed by rigid or non-rigid image-fusion and registration using (dedicated) ablation confirmation software packages [11,12].

As such, multiple periprocedural tools, i.e., stereotactic navigation and real-time image fusion, have found their way into clinical daily practice [13,14]. One of these helpful tools is the administration of small doses of intra-arterial intrahepatic contrast agent (mixed bolus of contrast and saline—may vary per institute) via a catheter placed into the hepatic artery via the groin, known as CT hepatic arteriography (CTHA) [15]. Although this procedure has historically been described for diagnostic purposes [16,17], it has recently been demonstrated to be a promising technique that improves tumor and surrounding vascular structure(s) conspicuity, needle targeting, and real-time ablation zone visualization [15]. These findings resulted in increased local disease control and superior LTPFS compared to conventional CT fluoroscopy guidance [15]. By using CTHA-guidance as an alternative guidance tool, more patients, for example, those with poorly visible lesions on ultrasound or conventional CT fluoroscopy, could become eligible for percutaneous thermal ablation [15,18]. For the detection of additional tumors or local tumor progression at the edge of the prior ablation zone (‘incomplete ring-sign’), CTHA was also found to be helpful [19].

In this pictorial essay, eight clinical cases from three institutions will be illustrated and discussed, demonstrating the added value of CTHA-guidance in percutaneous liver tumor ablation.

## 2. Cases

**Figure 1:**
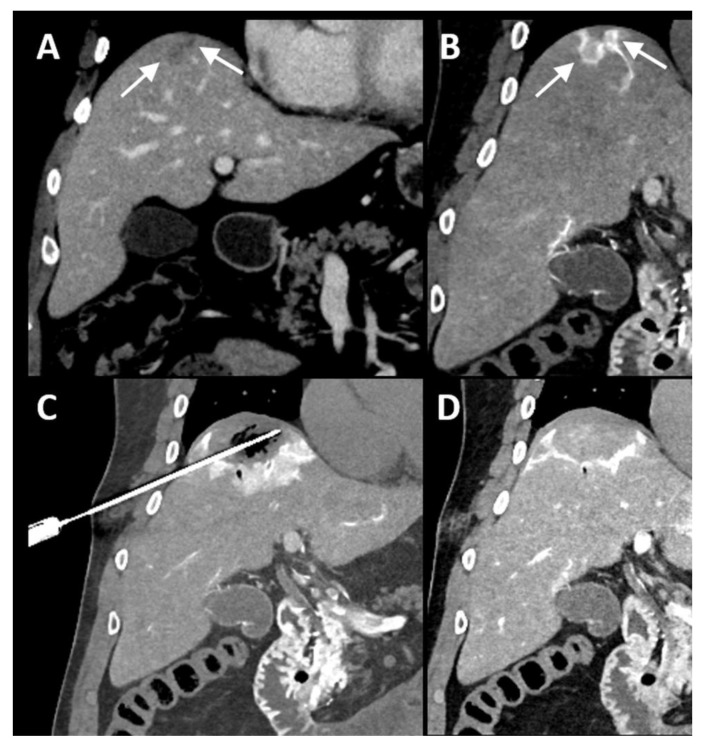
Identification of colorectal liver metastasis. Two adjacent colorectal liver metastases (arrows) in the hepatic dome, hardly visible on diagnostic CT-imaging in portal venous phase (**A**). After catheterization of the common hepatic artery, the lesions (arrows) became clearly visible as enhancing rings after injection of 20 mL of contrast agent (Visipaque™, iodixanol, 320 mg Iodine/mL, GE Healthcare Inc., Marlborough, MA, USA) just prior to probe placement (**B**). At the early end of microwave delivery (65 W, 7 min), 15 mL of contrast agent was injected, confirming adequate ablation coverage of both tumors (**C**), and another 20 mL was injected after complete ablation to assess the ablation zone and potential complications (**D**).

**Figure 2:**
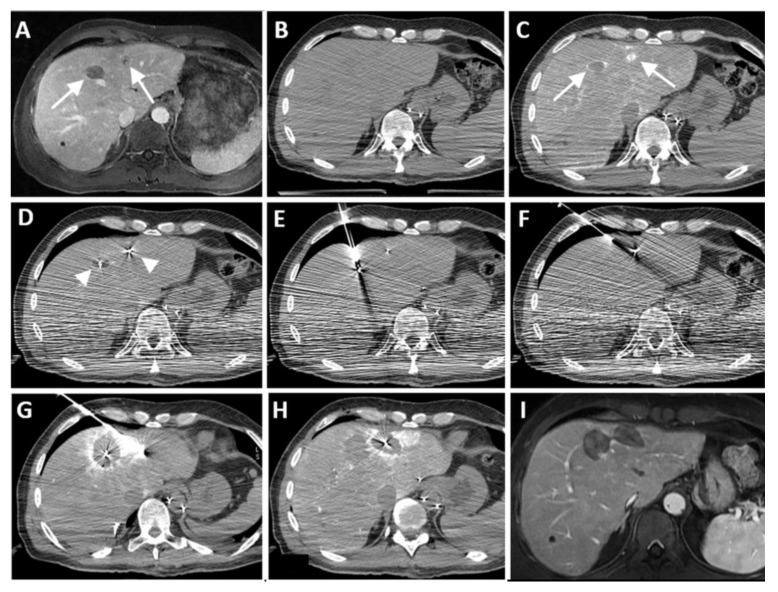
Identification of colorectal liver metastases. Two colorectal liver metastases (arrows) visible on diagnostic contrast-enhanced MRIs (**A**). During the procedure, the lesions were not seen on non-enhanced CT (**B**). After catheterization of the common hepatic artery, the lesions (arrows) became clearly visible as enhancing rings after the injection of 4 mL Xenetix 300^®^ contrast (Iobitridol 300 mg Iodine/mL, Guerbet, Villepinte, France) (**C**). Additionally, in this case, 0.018” coils (arrow heads) (Tornado, Cook Medical) have been inserted via a 22 G Chiba needle (Cook Medical, Bloomington, IN, USA) in the middle of the lesions as a fiducial marker and to facilitate post-ablation safety margin assessment (**D**) [22]. A carboxypneumothorax was being created prior to the actual RFA procedure (**E**,**F**) (Cool-tip™, Medtronic-Covidien, Boulder, CO, USA). Another 4 mL contrast was injected after each ablation to assess the created ablation zone (**G**,**H**). A follow-up MRI after two months showed no signs of local tumor progression (**I**).

**Figure 3:**
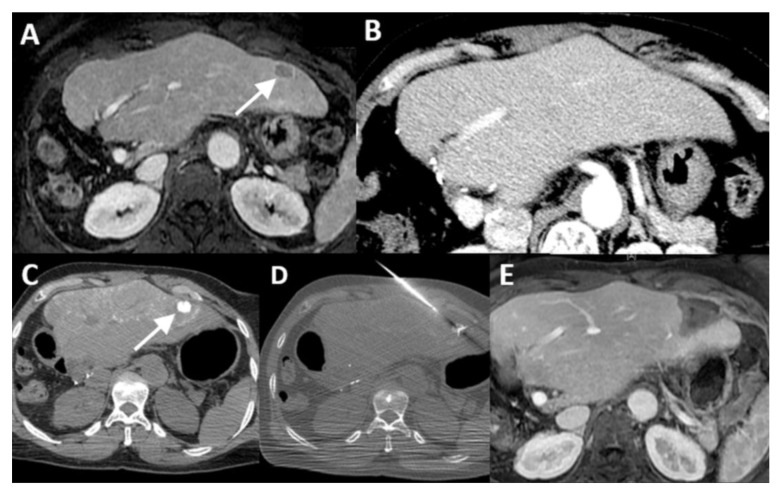
Visualization of the lesion by Lipiodol^®^ Ultra Fluid contrast agent. Solitary colorectal liver metastasis (arrow) in the left liver lobe is visible on diagnostic MRI (**A**), but not on the intraprocedural, intravenous contrast-enhanced CT in the late arterial to early portal venous phase (**B**). After selective catheterization of the left hepatic artery and administration of intra-arterial Lipiodol^®^ Ultra Fluid contrast agent (Guerbet, Villepinte, France), the lesion (arrow) became clearly visible as an enhancing nodule on non-enhanced CT imaging (**C**). Percutaneous RFA (Cool-tip™, Medtronic-Covidien, Boulder, CO, USA) was performed successfully (**D**). Follow-up imaging showed no signs of local tumor progression (**E**).

**Figure 4:**
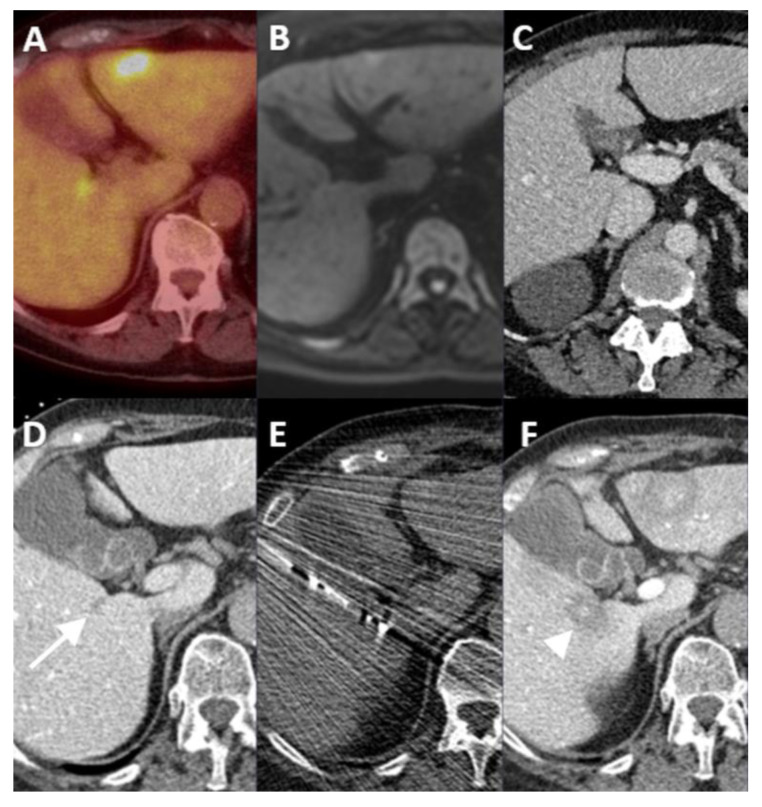
Visualization of a ‘vanished’ lesion after downstaging chemotherapy. Diagnostic ^18^F-FDG PET-CT showed two colorectal liver metastases ((**A**), segment III and V). Chemoradiation was given to pretreat the rectum tumor. The tumor in segment V ‘vanished’ on post-chemoradiation MRI ((**B**), diffusion-weight imaging, b-800) and intravenous contrast-enhanced CT (**C**). The patient qualified for local ablative treatment as the tumor in segment III was still visible. After catheterization of the common hepatic artery (to visualize both liver lobes) followed by the administration of 20 mL intra-arterial Xenetix 300^®^ (1:1 mixed bolus of saline and contrast agent; Iobitridol 300 mg Iodine/mL, Guerbet, Villepinte, France), the lesion (arrow) became clearly visible as a hypodense lesion of 9 mm in the portal venous phase (scanned at 22 s) (**D**). Percutaneous microwave ablation (Emprint™ Microwave Ablation System, Medtronic-Covidien, Boulder, CO, USA) was successfully performed (**E**), and the tumor was circumferentially covered by the ablation zone (arrow head) (**F**).

**Figure 5:**
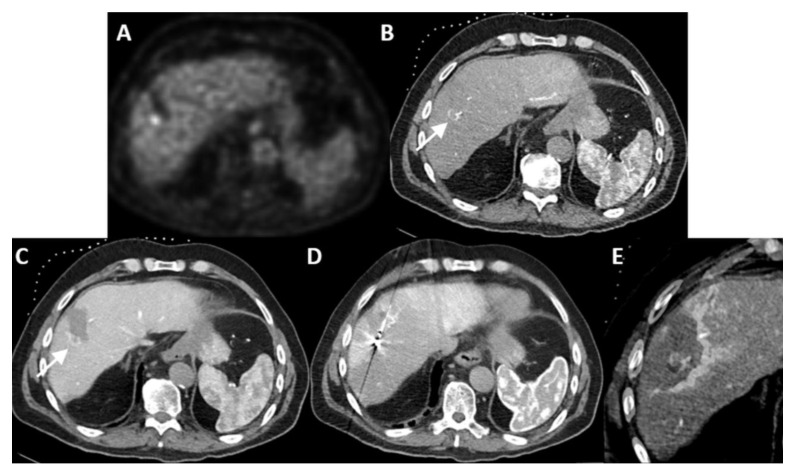
Differentiation between residual or recurring tumor tissue and non-enhancing scar tissue. Follow-up ^18^F-FDG PET-CT showed focal FDG uptake at the edge of the ablation zone in segment VIII (**A**). Catheterization of the coeliac trunk and intra-arterial administration of 40 mL Xenetix 300^®^ (1:1 mixed bolus of saline and contrast agent; Iobitridol 300 mg Iodine/mL, Guerbet, Villepinte, France) showed a typical incomplete enhancing ring (arrow), identified at the interface with the post-ablation scar tissue ((**B**), arterial phase at 7 s; (**C**), portal venous phase at 22 s). Intraprocedural verification of the microwave needle (Emprint™ Microwave Ablation System, Medtronic-Covidien, Boulder, CO, USA) (**D**). Overlay of pre- and postprocedural CT images (image fusion) showed complete coverage of the tumor by the ablation zone (**E**).

**Figure 6:**
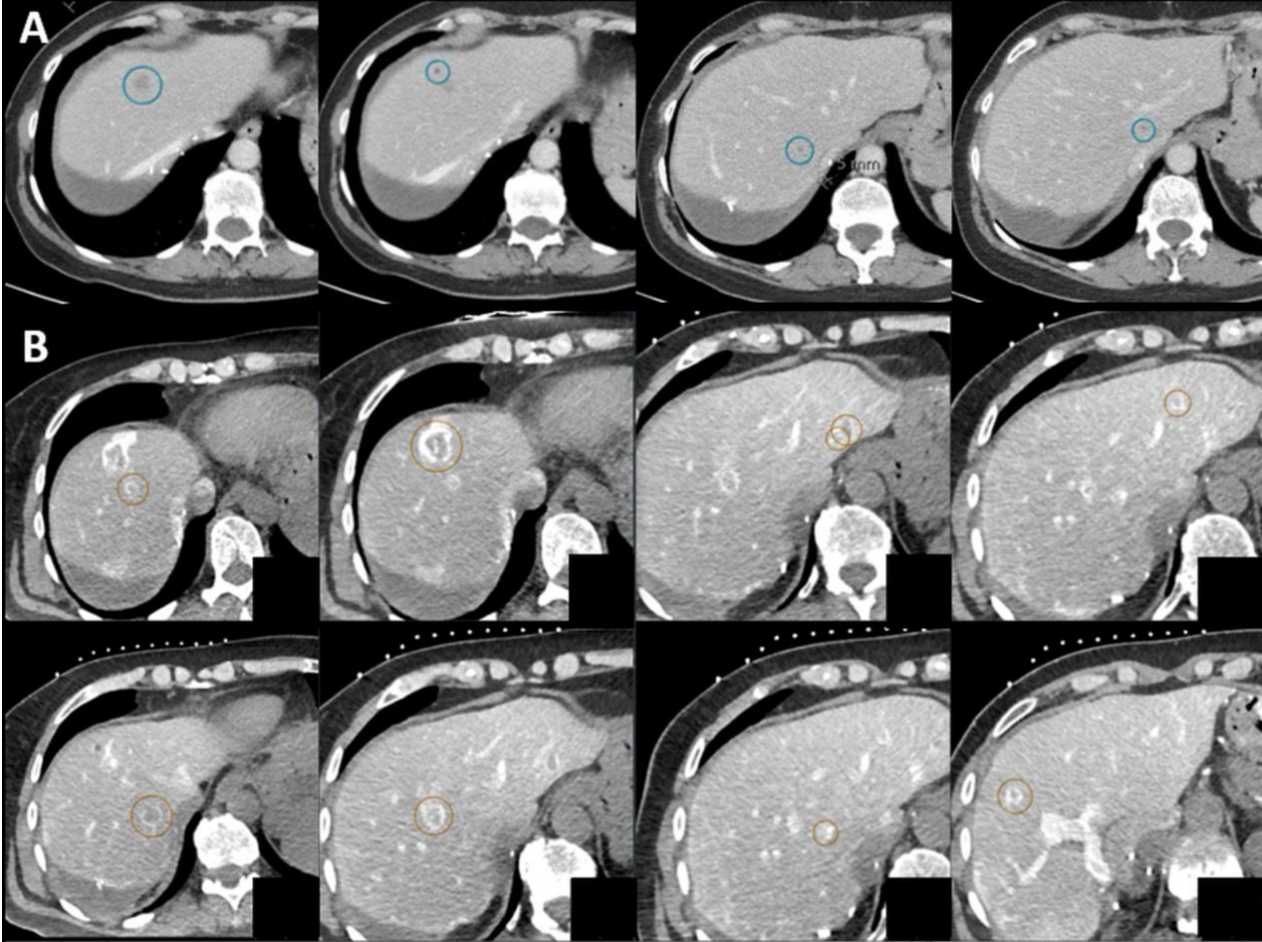
Detection of additional lesions during the procedure. Progressive disease after partial right-sided hepatectomy manifesting in four tumors (blue circle) seen on follow-up contrast-enhanced CT (**A**). The percutaneous microwave procedure was planned within three weeks after the follow-up scan. During the procedure, after catheterization of the proximal common hepatic artery administration of 40 mL Xenetix 300^®^ (1:1 mixed bolus of saline and contrast agent; Iobitridol 300 mg Iodine/mL, Guerbet, Villepinte, France), at least five additional ring-enhancing lesions (red circles) were found throughout the liver (**B**). Due to the extensiveness of the disease, the procedure was terminated. One day after the procedure, the additional lesions were confirmed to be metastases showing diffusion restriction on MRI.

**Figure 7:**
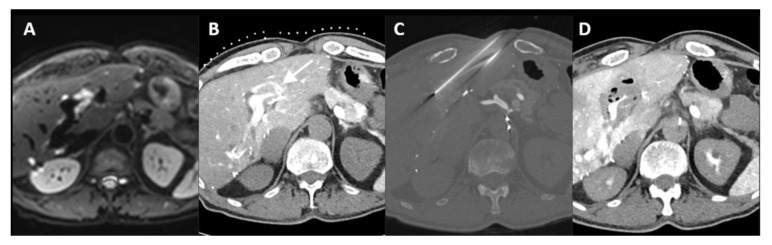
Identification of surrounding vascular structures for safety reasons. Progressive disease with five colorectal liver metastases, of which one was located in segment II/III surrounding a branch of the portal vein ((**A**), diffusion restriction on MRI). After the administration of intra-arterial contrast, 20 mL Xenetix 300^®^ (1:1 mixed bolus of saline and contrast agent; Iobitridol 300 mg Iodine/mL, Guerbet, Villepinte, France) in the proximal common hepatic artery, the tumor (arrow) became clearly visible (**B**). In order to preserve the vascular structure, irreversible electroporation (NanoKnife system under ECG-gating; AccuSync model 72; AngioDynamics, Latham, NY, USA) was performed by using four electrodes (20 mm exposure length, sequential pulses 10–90) (**C**). Postprocedural image fusion showed no complications and sufficient ablation margins with tailoring of the portal vein branch’ (**D**).

**Figure 8:**
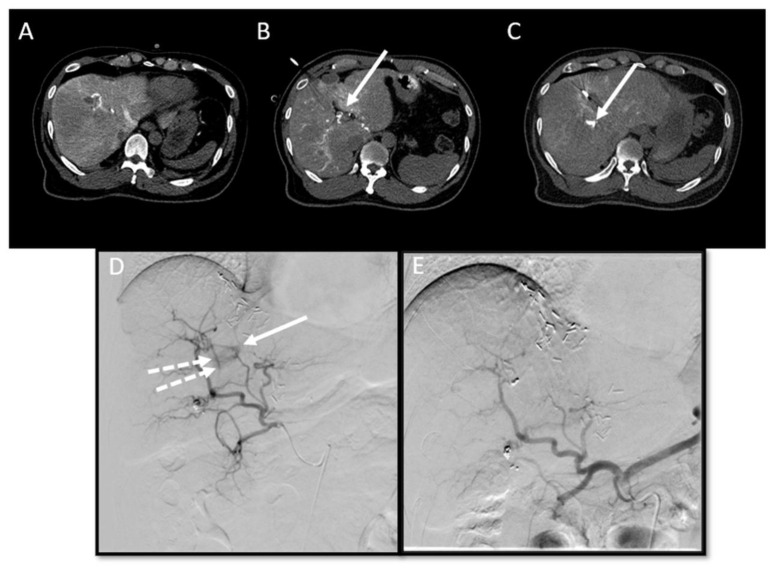
Critical care management: detection of post-ablation intraparenchymal hemorrhage and haemobilia. Probe-induced hepatic hemorrhage is seen in 0.7% of the patients [23]. Local tumor progression in segment 8 after parenchymal-sparing hepatectomy and intra-operative and percutaneous microwave ablations. After administration of 20 mL intra-arterial contrast (Visipaque™, iodixanol, 320 mg Iodine/mL, GE Healthcare Inc., Marlborough, MA, USA) in the common hepatic artery, the recurring tumor tissue became visible by a clear ‘incomplete ring sign’ adjacent to the prior ablation zone ((**A**), CTHA with a combined hybrid angio-CT system) [21]. Intraprocedural CTHA imaging depicted an incidental contrast agent within the bile duct (arrow), consistent with hemobilia (**B**). In addition, CTHA also depicted a small intraparenchymal contrast agent deposition (arrow), consistent with intraparenchymal hemorrhage (**C**). Immediately after the completion of ablation (Emprint™ Microwave Ablation System, Medtronic-Covidien, Boulder, CO, USA), digital subtraction angiography (DSA; contrast agent, Omnipaque 300, GE Healthcare, Cork, Ireland) of the common hepatic artery was performed, disclosing contrast pooling from the A4 branch ((**D**), arrow), as well as hemobilia ((**D**), dashed arrows), for which a combination of glue, Lipiodol^®^ Ultra Fluid contrast agent (Guerbet, Villepinte, France) and coils was used. Post-embolization DSA of the A4 and A8 branches depicted bleeding cessation (**E**).

This pictorial review was conducted at the interventional radiology departments of the Amsterdam University Medical Center location VUmc (Amsterdam, The Netherlands), the University of Texas MD Anderson Cancer Center (Houston, TX, USA), the Institut de Cancérologie Gustave Roussy (Villejuif, France), and the University Medical Center Utrecht (Utrecht, The Netherlands), all tertiary referral institutions for hepatobiliary and gastrointestinal malignancies. Disease-specific parameters and imaging data were collected and reported anonymously, not requiring ethical approval from each of the contributing institutions. The CTHA technique protocol has previously been described in detail by Puijk et al. and Van Tilborg et al. [15,18]. The type of intra-arterial catheter is physician-dependent; however, the following catheters for catheterization of the coeliac trunk and common/proper hepatic artery are regularly used in the contributing institutes; 4-French Cobra (Cordis Corp., Bridgewater, NJ, USA), 5-French Cobra (Cook, Inc., Bloomington, IN, USA), 5-French coeliac (Rosch hepatic; Cook, Bloomington, IN, USA), and 4-French SIM (Simmons, Merit Medical, Salt Lake City, UT, USA). The local CTHA procedure guidelines were similar between the Amsterdam UMC and Gustave Roussy Cancer Center (angiography system, Philips Azurion; Philips Healthcare, Best, The Netherlands; CT scanner, SOMATOM Sensation or Drive, Siemens Healthineers AG, Erlangen, Germany). In MD Anderson Cancer Center, ablations are either performed by conventional CTHA guidance or by using a combined hybrid angiography-CT system for the more recent cases (Nexaris Angio-CT, Siemens Healthineers AG, Erlangen, Germany) [20]. In Utrecht UMC, the ablation is performed using the C-arm Cone-beam CT within the angiography system (Philips Azurion; Philips Healthcare, Best, The Netherlands) [21]. Each case is accompanied by diagnostic imaging and intraprocedural CTHA images. The cases are presented in Figure 1, Figure 2, Figure 3, Figure 4, Figure 5, Figure 6, Figure 7 and Figure 8. Additional procedure-related details are shown in Table 1.

## 3. Discussion

CT hepatic arteriography guided liver ablation has recently been compared with conventional CT fluoroscopy guidance in liver tumor ablation and was found to be safe with superior LTPFS [15]. As supported by the provided clinical cases, the use of an intra-arterial contrast agent in the coeliac trunk, common or proper hepatic artery, or more selectively, in the hepatic artery contributes to increased delineation of liver lesions, more accurate ablation applicator placement, and superior coagulation necrosis visualization—all allowing for more precise ablation zones and wider circumferential safety margins.

The first two cases underline the importance of tumor visualization, especially for cases where tumors are hardly visible on diagnostic CT imaging in the portal venous phase or not visible on unenhanced imaging during the preprocedural planning scan (Figure 1 and Figure 2). Additionally, per local preferences, 0.018” coils can be inserted in the center of the tumor as a fiducial marker and to facilitate post-ablation safety margin assessment (Figure 2) [22]. An alternative to using solely iodine contrast agents, ethiodized oil (Lipiodol^®^, Guerbet, Villepinte, France) can be used for prolonged tumor visualization (Figure 3). Lipiodol^®^ has been used in transarterial chemoembolization, where it has been shown to be more densely retained within liver tumors than alternative water-in-oil emulsions when paired with selected drug(s) [24]. These oily features seem to facilitate transarterial catheter assisted ablation as well. Furthermore, CTHA guidance was found to be associated with a significantly smaller amount of contrast (88.4 mL) needed per procedure compared to conventional CT fluoroscopy guidance (131.0 mL; *p* < 0.001) [15]. Another advantage of transcatheter image-guided tumor ablation can be found in cases with significant therapeutic dilemmas—shrinkage of the tumor on ceMRI after downstaging chemotherapy, better known as ‘vanishing metastases’—which can be undertaken by the use of hepatic arteriography during the ablation procedure. This is underlined by Figure 4, which shows a tumor that disappeared on diffusion-weighted and post-contrast MRI images, while the tumor could be delineated after administering intra-arterial contrast during the ablation procedure [25]. As supported by Figure 5, previously reported results by Van Tilborg et al. underline that CTHA-guidance might also contribute to superior differentiation between viable residual or recurring tumor tissue and non-enhancing scar tissue (‘incomplete ring sign’), indicating LTP [18]. Another potential advantage has been previously published by Ohki et al., highlighting the ability to detect additional lesions during diagnostic workup [16]. Translating that into the therapeutic setting, additional lesions may be found during the ablation procedure after injecting intra-arterial contrast, as shown in Figure 6 and previously described by Van der Lei et al. [19]. The ease of immediately treating iatrogenic post-ablation hemorrhage is another advantage of having an intra-arterial catheter in position during the procedure, as shown by the presented case in Figure 8.

Possible complications related to catheter placement are active bleeding or a pseudo-aneurysm at the access site in the common femoral artery. Although these complications were not seen in the recently published study by Puijk et al. [15], active bleeding can be treated instantly (Figure 8). In our experience, most cases of active hemorrhage detected with (pump-injected) CTHA are minor hemorrhages that resolve within a few minutes.

Catheter placement is an additional procedure performed in the angio-suite, which entails marginal additional costs, which, in our opinion, is endurable when it comes to optimizing patient care. The additional costs and time required compared to ablation without CTHA can be reduced by performing the entire procedure (both catheterization and ablation) in the angio-suite. Although the catheter placement itself provides an extra negligible radiation dose to the patient, a dose-comparing study between a CTHA-guided and conventional CT fluoroscopy guided procedure has never been executed. Theoretically, better lesion conspicuity with CTHA might allow fewer needle repositionings with fewer single-shot CT images, leading to lower radiation exposure. In departments where angio-CT systems are not available, catheter placement does increase the number of bed-to-bed movements, which can induce tip dislocation. If this occurs, the tip can be left in the abdominal aorta at the level of the coeliac trunk to obtain an arteriogram (with higher dosages of contrast agent, i.e., 40 mL mixed contrast). Although arterial and portal venous phases may exhibit reduced tumor delineation when selective catheterization of the common or proper hepatic artery did not succeed, in the vast majority of cases, in our opinion, tip dislocation during bed-to-bed movements did not significantly compromise the images when higher dosages of contrast agents were administered. Extra bed-to-bed movements can be prevented either by using a mobile C-arm system in the CT suite, if suitable for the CT system, or by performing the entire ablation procedure in the angiography suite using a combined hybrid angiography-CT system [21,26,27,28].

Besides the advantages of transcatheter intra-arterial contrast administration, there are multiple other advances that have lately been developed and investigated in order to improve the success of thermal ablation procedures. In addition to the use of hepatic arteriography, segmentation and rigid and non-rigid co-registration in three dimensions all help to increase lesion detection, improve needle targeting, and thereby decrease residual unablated tumor rates and may shorten procedural time [11]. Proprietary ablation-confirmation software (i.e., Ablation-fit^TM^, v1.0, R.A.W. Srl, Milan, Italy) is one of the latest developments, offering automatic three-dimensional (3D) segmentation of the liver and intrahepatic blood vessels and semi-automatic, non-rigid co-registration of the target nodules and volumes of necrosis achieved by the ablation in order to assess the precision and completeness of the ablation volume [13,14,29]. Image fusion and navigation systems that combine multiple modalities have also been developed and are used with ever-increasing frequency for tumor targeting by real-time fusion guidance of US combined with preplanned CT images [30]. This fusion system allows for the visualization of the tumor and the needle position for target tumors that are undetectable with US alone. Furthermore, a novel technique where the tip of the RFA electrode includes electromagnetic tip tracking shows the exact tip location by electromagnetic position sensor in US-guided radiofrequency ablation [31]. Unfortunately, no difference in technical thermal ablation success was found, and the proposed benefit of electromagnetic tip tracking was not realized. Additionally, Taghavi and colleagues proposed a CT-based radiomics analysis before thermal ablation and are, to our knowledge, the first to enable a machine learning-based radiomics analysis to predict LTP in thermal ablation in patients with CRLM [32]. This preprocedural predictive model could help guide treatment decisions to reduce LTP as well as the detection of high risk lesions for LTP. Augmented reality is the newest development, with systems combining tumor tracking and navigation software with goggles showing the needle and the landmarks on the patient’s skin [33], ultimately leading to systems where real-time ultrasound images are also displayed in the goggles. These novel techniques, all focused on advanced imaging and innovative real-time image guidance techniques, and artificial intelligence instruments, are not yet able to substitute our currently available techniques and should be further explored and validated in future studies.

## 4. Conclusions

This pictorial review encompasses illustrative cases of CT hepatic angiography guidance in the percutaneous thermal ablation of primary or secondary liver cancer. This technique offers the ability of viable tumor tissue visualization, more precise targeting, and less needle repositioning, allowing for the ability to: (a) create an adequate circumferential safety margin around the initial lesion, (b) detect additional lesions during the procedure, (c) visualize surrounding vascular structures for safety reasons, (d) differentiate residual or recurring tumor tissue from non-enhancing scar tissue (‘incomplete ring sign’, indicating LTP), (e) identify vanishing lesions after downstaging chemotherapy, and (f) promptly deal with a potential probe-induced hemorrhage in the liver. Although at the cost of adding a minor intervention, CTHA-guided liver tumor ablation minimizes complications by combining both CTHA ablation and software-aided ablation margin assessment, which will undoubtedly improve local disease control and reduce the number of re-interventions needed. Future developments on real-time fusion imaging, volumetric assessment of the peri-ablational safety margin with biomechanical ablation software, needle and electromagnetic tracking devices, and machine learning radiomics and augmented reality tools could be of immense value for intraprocedural decision-making and could potentially positively impact LTP rates. We recommend adopting CTHA as a quality-improving guiding method within the (inter)national standards of practice.

## Figures and Tables

**Table 1:** Baseline information.

Case No.	Tumor Size (mm)	Catheter Tip Position	Amount and Type of Contrast per Injection	Ablation Device	Institute
1	20 mm	Common hepatic artery	15–20 mL Visipaque™	Emprint™ Microwave Ablation System, Medtronic-Covidien, Boulder, CO, USA	MD Anderson, Houston, TX, USA
2	20 and 7 mm	Common hepatic artery	4 mL Xenetix 300^®^	Cool-tip™ RFA Ablation Aystem, Medtronic-Covidien, Boulder, CO, USA	Gustave Roussy, Villejuif, France
3	12 mm	Left-sided hepatic artery	Not specified	Cool-tip™ RFA Ablation System	Gustave Roussy
4	9 mm	Common hepatic artery	10 mL Xenetix 300^®^	Emprint™ Microwave Ablation System	Amsterdam UMC, Amsterdam, The Netherlands
5	15 mm	Coeliac trunk	20 mL Xenetix 300^®^	Emprint™ Microwave Ablation System	Amsterdam UMC
6	Not applicable	Common hepatic artery	20 mL Xenetix 300^®^	Emprint™ Microwave Ablation System	Amsterdam UMC
7	Confluent	Common hepatic artery	10 mL Xenetix 300^®^	NanoKnife system under ECG-gating; AccuSync model 72, AngioDynamics, Latham, NY, USA	Amsterdam UMC
8	15 mm	Common hepatic artery	15–20 mL Visipaque™	Emprint™ Microwave Ablation System	MD Anderson
